# Fabrication of Nitrogen-Containing Micro-Expanding Graphite Composites from Waste Graphite Electrodes for Enhanced Lithium Storage

**DOI:** 10.3390/nano16080485

**Published:** 2026-04-19

**Authors:** Xu Fan, Zhuohan Lv, Hongyan Nan, Daoguang Teng, Baolin Xing, Peng Li

**Affiliations:** 1 State Key Laboratory of Critical Metals Beneficiation, Metallurgy and Purification, School of Chemical Engineering, Zhengzhou University, Zhengzhou 450001, China; fx2023@gs.zzu.edu.cn (X.F.); lvzhuohan@gs.zzu.edu.cn (Z.L.); nanhongyan@zzu.edu.cn (H.N.); teng_daoguang@zzu.edu.cn (D.T.); 2 The Key Lab of Critical Metals Minerals Supernormal Enrichment and Extraction, Ministry of Education, Zhengzhou University, Zhengzhou 450001, China; 3 College of Chemistry and Chemical Engineering, Henan Polytechnic University, Jiaozuo 454003, China; baolinxing@hpu.edu.cn

**Keywords:** waste graphite electrodes, nitrogen doping, micro-expanding graphite, lithium-ion battery anode, electrochemical performance

## Abstract

The large-scale generation of waste graphite not only poses environmental challenges but also provides an opportunity for resource recovery. This study proposes a sustainable strategy that utilizes the graphite cutting waste produced during the production of large graphite electrodes through chemical intercalation, microwave-assisted expansion, and in situ urea nitrogen doping techniques to prepare nitrogen-containing micro-expanded graphite (NMG) composite materials. Structural analysis reveals that the nitrogen-doped amorphous carbon layer formed on the expanded graphite (EG) matrix effectively suppresses excessive expansion while preserving its typical worm-like interlayer morphology and porous structure. XPS confirms successful nitrogen doping with predominant pyridinic-N configuration, introducing abundant defect sites and enhancing lithiophilicity. As an anode for LIBs, NMG delivers an exceptional initial discharge capacity of 1907.5 mAh g^−1^ at 20 mA g^−1^ and maintains 798.2 mAh g^−1^ after 50 cycles, nearly twice that of purified waste graphite (G). Remarkably, after 1000 cycles at 1 A g^−1^, it retains 650.4 mAh g^−1^ with 89.9% capacity retention, indicating an electrochemical activation process. Kinetic analysis reveals that the superior performance originates from synergistic diffusion-controlled intercalation and surface-dominated pseudocapacitance, with nitrogen-doped defect sites and hierarchical pore architecture promoting rapid ion/electron transport and surface faradaic reactions. This work demonstrates a viable pathway for value-added upcycling of waste graphite while providing insights into designing high-performance anodes through integrated defect engineering and heteroatom doping.

## 1. Introduction

The rapid development of the electric vehicle industry, coupled with the continuous miniaturization and functional upgrades of portable electronic devices, has driven a significant increase in global demand for high-energy-density and high-efficiency energy storage systems. Lithium-ion batteries (LIBs) have mitigated the intermittency of renewable energy sources and satisfy diverse market requirements, primarily owing to their superior energy density, long-term cycling performance, and high coulombic efficiency (CE) [1,2,3,4]. However, in order to further enhance the energy density, power density and long-term cycle stability to meet the increasing demands of next-generation LIBs, there are still many technical obstacles that need to be overcome [5,6,7,8].

As a new type of carbon material, expanded graphite (EG) has become a highly promising anode material for LIBs due to its unique structural configuration and increased interlayer spacing [9,10,11,12,13]. Firstly, the interlayer spacing is significantly increased by the expansion treatment, and a worm-like structure is formed while maintaining the ordered layered structure of graphite [14]. Secondly, the EG possesses a hierarchical honeycomb-like pore structure, characterized by the ordered arrangement of wrinkled graphene nanosheets [15,16]. Finally, the EG has an ultra-high specific surface area, due to the increasement of interlayer spacing [17]. These microstructures not only provide ideal channels for the transmission of lithium-ion, but also increase the active sites for lithium-ion. These structural characteristics enable EG to exhibit excellent electrochemical reaction kinetics performance, which significantly improves its reversible specific capacity and rate performance [18]. Deng et al. found that EG prepared from anthracite as the raw material exhibited excellent electrochemical performance when used as the negative electrode of lithium-ion batteries. After 300 cycles, it still maintained an extremely high Coulombic efficiency [19]. Gong et al. used EG prepared from the graphite negative electrode recovered from LIBs and found that it could still maintain a relatively high specific capacity after 1000 cycles [20]. Fu et al. carried out ball milling treatment on acidic graphite oxide (AGO) and polyaniline, and then conducted chemical expansion at a low temperature, resulting in a negative electrode material with excellent electrochemical properties [21].

As an anode material for LIBs, EG possesses outstanding electrical conductivity and structural stability, yet its practical commercial application in LIBs is still limited by numerous critical challenges. The most important thing is that the expansion of graphite oxide (GO) usually occurs in an instant, making it difficult to control the extent of the expansion [22]. Moreover, the excessively high specific surface area (SSA) of EG intensifies the secondary reactions at the electrode/electrolyte interface. This leads to a continuous depletion of electrolytes and causes the solid electrolyte interface (SEI) layer to grow continuously and unstably, thereby seriously compromising the long-term cycling stability of the battery [23,24]. To address this key scientific challenge, in this study, the GO precursor was prepared via a modified Hummers method. Compared with the conventional Hummers method, our protocol eliminates the use of NaNO_3_, and the entire preparation process is carried out under mild conditions, avoiding the low-temperature ice-bath pretreatment and high-temperature reaction termination steps required in the traditional method. On this basis, we develop a novel one-step microwave-assisted expansion strategy, in which urea is introduced simultaneously as both a nitrogen source and a structure regulator. With this method, we successfully fabricate nitrogen-doped micro-EG (NMG) with a well-tuned and stable interlayer structure, which serves as a promising anode candidate for high-performance lithium-ion energy storage applications. This study systematically investigated the influence of urea doping on the microstructure evolution of the material, and revealed the structural-activity relationship between its structure and electrochemical properties (including specific capacity, rate performance and cycle life). These research results provide a theoretical basis and practical approach for the development of high-performance anode materials for LIBs and the exploration of application directions for waste graphite.

## 2. Experiments

### 2.1. Material Preparation

Purified waste graphite (denoted by G) with a carbon content exceeding 99 wt.% was reclaimed from spent graphite electrodes (Figure S1). First, 1.0 g of the G was dispersed in 23 mL of concentrated sulfuric acid (H_2_SO_4_, 98 wt.%) under magnetic stirring at 25 °C. Subsequently, 3.0 g of potassium permanganate (KMnO_4_) was slowly added to the suspension, and the reaction was allowed to proceed for 10 min. The temperature was then elevated to 35 °C and maintained for an additional 5 min. Next, 140 mL of deionized water was added into batches, followed by the addition of 10 mL of hydrogen peroxide (H_2_O_2_, 30 wt.%) to reduce the excess oxidant [25]. The resulting mixture was allowed to stand for 24 h and then ultrasonicated for 15 min. The products were collected by filtration and repeatedly washed with deionized water until the pH of the filtrate reached 7, yielding graphene oxide (GO).

**Figure 1:**
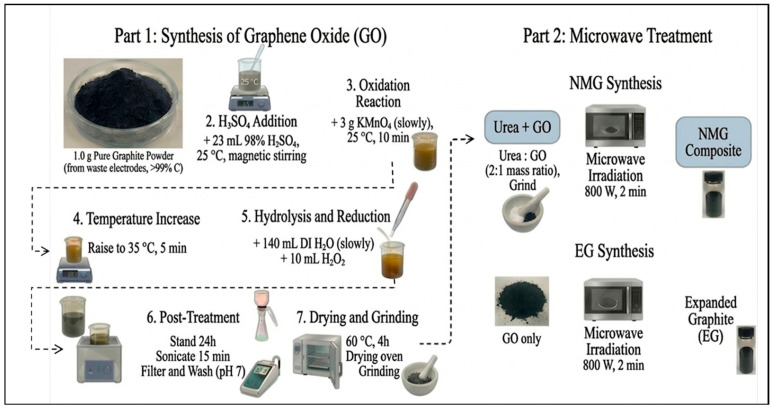
Flow charts of the EG and the NMG preparation.

The obtained GO was dried at 60 °C for 4 h and subsequently ground into a fine powder. To prepare the NMG composite, urea and the GO were thoroughly mixed and ground at a mass ratio of 2:1. The mixture was then subjected to microwave irradiation at 800 W for 2 min. For comparison, the EG was prepared under identical microwave conditions using pure GO without the addition of urea (Figure 1).

### 2.2. Material Characterization

The microstructure and elemental distribution of the samples were analyzed using an electron probe microanalyzer (EPMA-8050G, Shimadzu Corp., Kyoto, Japan). The crystalline phases of the materials were characterized by X-ray diffraction (XRD) on a Bruker D8 Advance diffractometer (Bruker AXS SE, Karlsruhe, Germany) equipped with Cu-K*α*_1_ radiation (λ = 1.5406 Å). The XRD patterns were recorded at a scanning speed of 5°/min across the 5~90° range. Fourier transform infrared (FTIR) spectra were recorded over the frequency range of 4000~500 cm^−1^ using a Frontier spectrometer (PerkinElmer, Waltham, MA, USA). The composition and molecular structure of the materials were characterized using a LabRAM HR Evolution Raman spectrometer (HORIBA, Paris, France) with a 532 nm laser excitation over a scanning range of 50~3500 cm^−1^. The X-ray photoelectron spectroscopy (XPS) was performed using a Thermo Scientific K-Alpha spectrometer (Thermo Fisher Scientific, Waltham, MA, USA) to characterize the elemental composition, surface functional groups, and chemical valence states of the samples. The specific surface area and pore structure of the samples were characterized by nitrogen adsorption–desorption isotherms at 77 K using an automated surface area and porosity analyzer (ASAP 2460, Micromeritics, Norcross, GA, USA).

### 2.3. Electrochemical Measurement

The electrochemical performance was evaluated using CR2032-type coin cells. The working electrode was prepared by dispersing the active material, acetylene black (conductive agent), and polyvinylidene fluoride (PVDF, binder) in N-methylpyrrolidone (NMP) at a mass ratio of 80:10:10. The resulting homogeneous slurry was cast onto high-purity copper foil and dried in a vacuum oven at 110 °C for 12 h. The mass of the active material was approximately 0.8 mg. The dried electrode film was then punched into circular disks with a diameter of 12 mm, and the mass loading of the active substance was precisely recorded. The assembly of the button batteries was performed in an argon-filled glove box with moisture and oxygen content strictly maintained below 0.01 ppm. A pure lithium metal sheet was utilized as the counter electrode. The electrolyte comprised a 1.0 M solution of LiPF_6_ in a 1:1 (*v*/*v*) mixture of ethylene carbonate (EC) and diethyl carbonate (DEC). For each sample, four coin cells were assembled under identical conditions. Among them, two cells with the smallest performance deviation were selected, and the larger value from these two was adopted as the final representative electrochemical performance.

Galvanostatic charge–discharge (GCD) tests, including rate capability and long-term cycling performance, were conducted using a LAND battery testing system (CT3002, Wuhan, China) within a voltage range of 0.01-3.0 V (vs. Li/Li^+^) at room temperature. Cyclic voltammetry (CV) measurements were performed on a CHI760e electrochemical workstation (Chenhua, Shanghai, China) over the same potential range at scan rates varying from 0.1 to 10 mV s^−1^. Electrochemical impedance spectroscopy (EIS) was also carried out on the CHI760e workstation over a frequency range of 10 kHz to 0.1 Hz with an AC amplitude of 5 mV. The obtained EIS data were fitted and analyzed using ZSimpWin version 3.50 software. All electrochemical tests were carried out at room temperature.

The obtained CV curves were fitted, and the Dunn Formula (1) [26] was applied to determine the kinetic behavior of electrode materials based on current–response relationships under different scan rates, quantitatively calculating the contribution ratio of the dominant electrochemical storage process to capacitance. Parameters *a* and *b* in Formula (2) are adjustable. According to Formula (3), *K*_1_ and *K*_2_ values were fitted, while Formula (4) was used to calculate capacitance contributions, where *K*_1_*v* represents the capacitance contribution current, and *K*_2_*v*^0.5^ denotes the diffusion-controlled current.

(1) $$i=kv^{b}$$

(2) logi=blogv+loga

(3) $$\frac{i}{v^{0.5}}=K_{1}v^{0.5}+K_{2}$$

(4) $$i=K_{1}v+K_{2}v^{0.5}$$

To gain deeper insight into the electrochemical kinetics, the relationship between peak current (*i*) and scan rate (*v*) was analyzed using the power law relationship: logi=blogv+loga, where *b* is a critical parameter that characterizes the charge storage mechanism. A b-value of 0.5 signifies an ideal diffusion-controlled intercalation process, while a b-value of 1.0 corresponds to a purely surface-controlled capacitive process. When *b* falls between 0.5 and 1.0, it reflects a mixed control mechanism involving both diffusion-limited and surface-controlled contributions.

## 3. Results and Discussion

### 3.1. Morphology, Structure and Composition of the NMG

#### 3.1.1. Morphology and Pore Structure

**Figure 2:**
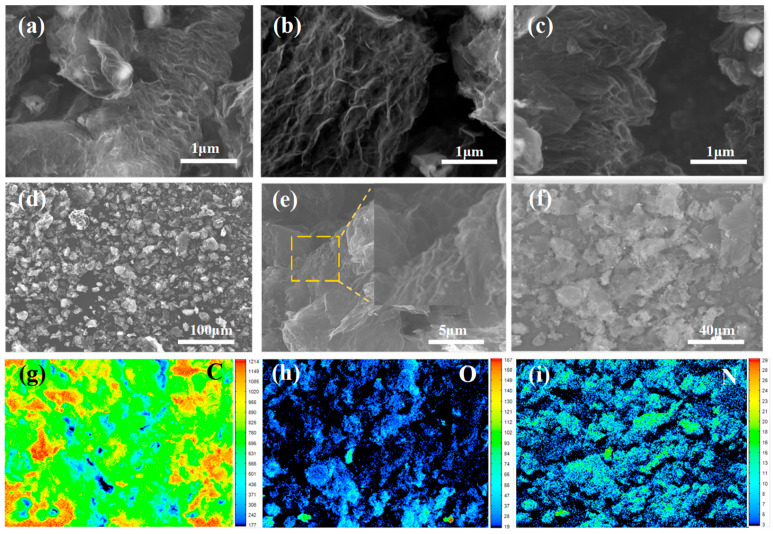
EPMA images of (**a**–**c**) EG and (**d**–**f**) NMG hybrids, and the mapping of (**g**) carbon, (**h**) oxygen and (**i**) nitrogen in NMG hybrids.

The NMG composite is constructed from an EG matrix, whose surface is modified by a nitrogen-rich carbon layer derived from the in-situ carbonization of urea. During the microwave irradiation, the rapid decomposition and carbonization of urea serve as an effective nitrogen source, facilitating the incorporation of nitrogen atoms into the graphitic lattice and their attachment onto the EG framework [27]. Compared with the significantly increased layer spacing and worm-like structure in the EG (Figure 2a–c), the NMG (Figure 2d–f) exhibited a notably reduced degree of expansion while still preserving the characteristic worm-like network structure typical of EG [23]. This constrained expansion is attributed to the formation of a rigid nitrogen-containing carbon layer on the NMG surface, which effectively prevents excessive exfoliation during microwave treatment (Figure 2e). Moreover, the EDS mapping reveals a uniform distribution of nitrogen throughout the hybrid material (Figure 2i), confirming successful and homogeneous nitrogen doping.

**Figure 3:**
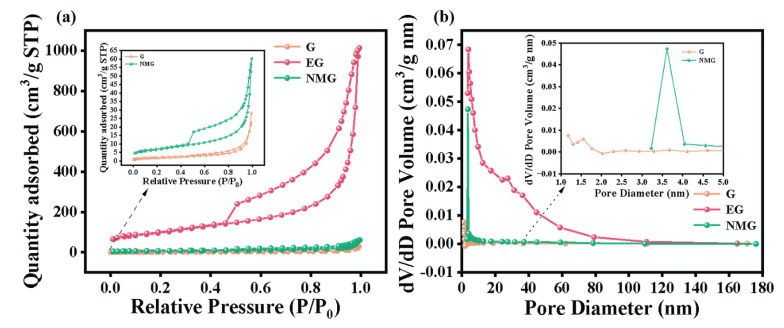
(**a**) Nitrogen adsorption–desorption and (**b**) pore size distribution of G, EG and NMG.

The porous structure of the as-prepared materials was further characterized by nitrogen adsorption–desorption measurements. As shown in Figure 3a, the G sample presented a typical Type III isotherm with a concave profile and no obvious inflection points, which is a well-recognized feature of non-porous or macroporous materials with weak adsorbent–adsorbate interactions. In contrast, both of the EG and the NMG exhibited typical Type IV isotherm characteristics. At low relative pressures (*P*/*P*_0_), the two samples showed limited nitrogen adsorption capacity, while a clear inflection point and a well-defined hysteresis loop emerged with increasing relative pressure. This discrepancy in isotherm features between EG/NMG and G can mainly be ascribed to the expanded interlayer structure of the modified graphite materials [5,28].

The corresponding SSA and pore volume data are presented in Figure 3b. The G material possesses an extremely low SSA of only 6.39 m^2^ g^−1^ and a negligible pore volume of 0.04 cm^3^ g^−1^, consistent with its dense, non-porous structure. Following microwave-assisted expansion, the SSA and pore volume of the EG increase dramatically to 347.51 m^2^ g^−1^ and 1.57 cm^3^ g^−1^, respectively, confirming substantial interlayer expansion and the creation of extensive porosity. In contrast, the NMG composite exhibits an intermediate level of 23.45 m^2^ g^−1^ and 0.09 cm^3^ g^−1^. As can be seen from the inset of Figure 3b, the pore size distribution curve exhibits a sharp peak in the range of 3–4 nm. Based on the desorption branch of the isotherm, the hysteresis loop closure occurring at a relative pressure of approximately 0.45 is the cause of the formation of this peak, thereby resulting in a deviation in the characterization of the actual intrinsic pore structure of the material. Nevertheless, the combined results of the material’s specific surface area and isotherm type still confirm that the introduction of urea not only inhibited the excessive expansion of GO, but also effectively retained abundant defect sites. This modulated layered structure is crucial for optimizing electrochemical performance. The intermediate surface area and pore volume of NMG establish a synergistic balance between providing sufficient electrochemically active surface area and maintaining robust structural integrity, which is a key requirement for achieving high specific capacity and long-term cycle life [29].

#### 3.1.2. Surface Crystal Phase Structure

**Figure 4:**
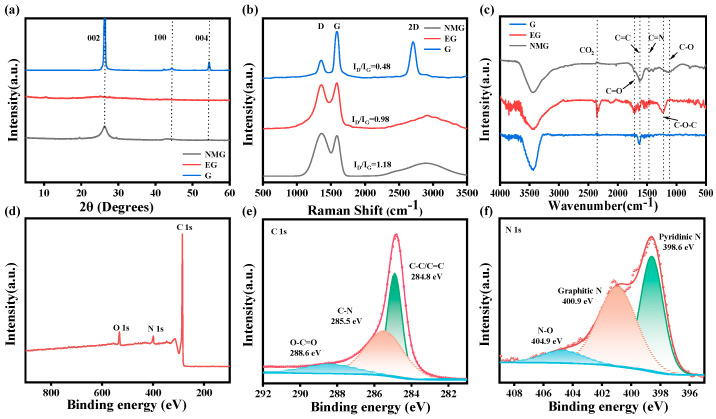
(**a**) XRD, (**b**) Raman, and (**c**) infrared spectra of NMG, EG, and G. (**d**) XPS spectra of NMG, and the (**e**) C 1s fitting and (**f**) N 1s fitting of NMG.

As shown in Figure 4a, the (002) diffraction peaks of both of the NMG and the EG are significantly weakened relative to that of the G. This attenuation indicates effective exfoliation of the graphite layers, leading to an increased interlayer spacing (d_002_) and a significant reduction in stacking thickness along the c-axis direction, consistent with successful structural expansion [30,31,32]. Notably, the XRD pattern of EG displays an additional diffraction peak at approximately 10.5°, corresponding to the (001) reflection of the residual GO. This suggests that the thermal exfoliation during the microwave treatment process was incomplete, leaving unreacted precursors in localized regions.

#### 3.1.3. Defect and Carbon Structure

Raman spectroscopy provides further insight into these structural transformations (Figure 4b). All three samples exhibit characteristic D and G bands at approximately 1351 cm^−1^ and 1591 cm^−1^, respectively. However, the 2D band, representing the layered graphite structure, appeared as a broad peak in NMG. The substantial decrease in layer number resulting from microwave-induced exfoliation leads to the formation of single- or few-layer configurations. This disruption of interlayer stacking is directly reflected in the evolution of the 2D band, whose broadening serves as compelling evidence for the presence of few-layer graphene or disordered stacked structures [33].

As further illustrated in Figure 4b, the I_D_/I_G_ intensity ratio of the EG and the NMG were significantly higher than that of the G, confirming a substantial increase in structural disorder following microwave expansion. Notably, the I_D_/I_G_ ratio of the NMG composite reaches to 1.18, indicating that its framework is predominantly composed of amorphous carbon. This structural evolution stems from the rapid thermal decomposition of urea during microwave irradiation and the subsequent in situ carbonization process. The resulting nitrogen-doped amorphous carbon serves a dual function: it effectively suppresses excessive growth of graphite layers while simultaneously introducing a high density of defect sites, which provide additional pathways and active sites for lithium-ion intercalation [34]. Beyond these chemical modifications, the honeycomb-like hierarchical pore structure retained in the NMG composite serves as an efficient transport network for lithium-ions (Figure 2), significantly facilitating their diffusion. Collectively, these synergistic structural features, including nitrogen-doped amorphous carbon, abundant defects, and interconnected porosity, underpin the outstanding electrochemical performance of NMG, particularly its superior rate capability and long-term cycling stability [15].

#### 3.1.4. Surface Composition and Chemical State

As presented in Figure 4c, both of the EG and the NMG exhibited two moderately intense characteristic absorption peaks at 1720 cm^−1^ and 1230 cm^−1^ corresponding to C=O and C-O bonds, respectively. The presence of these oxygen-containing functional groups not only enhances the wettability between the electrolyte and electrode surface but also provides additional active sites for lithium-ion adsorption, thereby contributing to improved electrochemical performance [35]. The XPS survey spectra confirms the successful integration of nitrogen into the NMG framework, as evidenced by the distinct N 1s peak (Figure 4d). High-resolution C 1s and N 1s spectra (Figure 4e,f) reveal that nitrogen atoms are incorporated both on the surface and within the lattice of the NMG, primarily existing as graphite nitrogen and pyridine nitrogen. The total nitrogen content in the NMG material is 5.3 wt.%. Among them, the proportion of graphite nitrogen and pyridine nitrogen are 46.3 at.% and 40.2 at.% respectively. Specifically, the introduction of pyridine nitrogen creates defect sites with localized high electron density, which significantly enhances the chemical adsorption capacity of lithium-ion and promotes interface charge transfer kinetics. This structural modification effectively reduces the activation energy for lithium-ion insertion, endowing the NMG with superior electrochemical performance relative to undoped or less-doped counterparts [36,37].

### 3.2. Electrochemical Properties

#### 3.2.1. Cyclic Voltammetry Behavior

**Figure 5:**
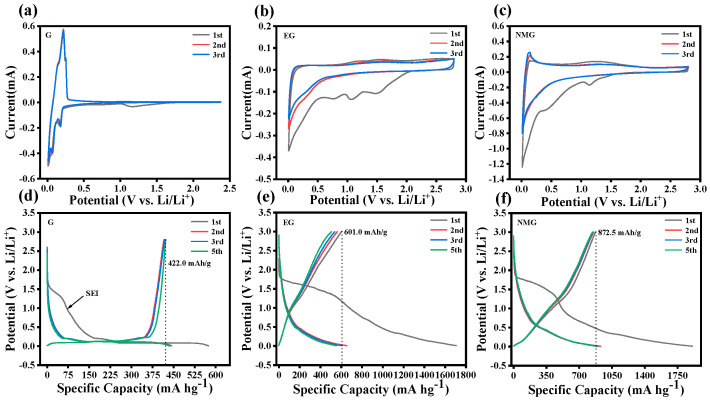
(**a**–**c**) CV curves of G, EG, and NMG at a scanning rate of 0.1 mV s^−1^ in the initial three cycles; (**d**–**f**) Initial five charge–discharge cycle curves of G, EG, and NMG at 20 mA g^−1^.

Figure 5a–c present the CV curves of the three materials recorded over the first three cycles at a scan rate of 0.1 mV s^−1^. During the first cathodic scan, all samples exhibit a distinct reduction peak at approximately 1.2 V, which disappears in subsequent cycles. This irreversible feature is attributed to the formation of the SEI film during the initial lithiation process [12,18,38]. In subsequent cycles, the CV curves show excellent overlap, indicating good electrochemical reversibility. Notably, the CV behavior differs significantly between the G and the expanded derivatives. As shown in Figure 5a, the G exhibits sharp oxidation–reduction peaks at fixed potentials, which are characteristic of typical battery-type energy storage behavior governed by diffusion-controlled intercalation processes [39]. In contrast, the CV curves of the EG and the NMG (Figure 5b,c) display a broad, approximately rectangular distribution with extremely narrow peak potential separations, suggesting a predominantly pseudocapacitive charge storage mechanism [29]. This transition in energy storage behavior arises from the oxygen-containing functional groups retained on the EG and NMG surfaces following microwave expansion [5,29,40]. Furthermore, in the case of the NMG, the lithiophilic nitrogen atoms introduced by urea decomposition actively participate in rapid faradaic reactions at the electrode surface, further enhancing the pseudocapacitive contribution. This fundamental shift in lithium storage mechanism, from diffusion-controlled intercalation in the G to surface-dominated pseudocapacitance in the NMG, constitutes the key rationale underlying the superior rate performance and high specific capacity exhibited by the nitrogen-containing composite.

#### 3.2.2. Galvanostatic Charge–Discharge Performance

Figure 5d–f present the GCD profiles of the three samples for the first five cycles at a current density of 20 mA g^−1^. As summarized in Table 1, the NMG composite exhibits remarkable electrochemical performance, delivering an initial discharge capacity of 1907.5 mAh g^−1^ and a charging capacity of 872.5 mAh g^−1^. These values substantially outperform those of pristine G, which shows only 575.5 mAh g^−1^ and 422.2 mAh g^−1^, respectively. This substantial capacity enhancement underscores the effectiveness of the nitrogen-doped micro-expanded architecture in promoting lithium storage.

However, the initial coulombic efficiency (ICE) of NMG is approximately 45.7%, significantly lower than that of the G. This trade-off between enhanced capacity and reduced ICE is primarily attributed to three interconnected factors. The expanded interlayer spacing provides additional intercalation lithium storage sites, the nitrogen-/oxygen-containing heteroatoms offer abundant pseudocapacitive active sites, and the moderate specific surface area brings extra surface adsorption capacity. These factors collectively contribute to the ultra-high initial specific capacity of the NMG. Although the specific surface area of the NMG is lower than that of the EG, it is still significantly higher than that of the G. The abundant electrode–electrolyte interface inevitably triggers irreversible electrolyte decomposition during the first discharge process, and the formation of a stable SEI layer consumes a large amount of active lithium ions, which is the dominant source of irreversible capacity loss. The abundant nitrogen-containing and oxygen-containing functional groups in the NMG undergo irreversible reduction reactions during the first discharge, and these active sites irreversibly trap partial lithium ions, which cannot be deintercalated during the subsequent charging process, resulting in additional irreversible capacity loss. During the first deep discharge, a small amount of lithium ions will be irreversibly trapped in the defect sites of the expanded interlayer structure and cannot be released, which also contributes to the irreversible capacity loss [23,24,41]. Despite the low ICE, the CE rapidly of the NMG recovers rapidly in subsequent cycles, reaching 91.9% in the second cycle and further increasing to 95.8% by the fifth cycle. Moreover, the NMG maintains 96.6% of its initial reversible capacity after five cycles.

In contrast, the specific capacity of EG shows a sharp decline trend. It dropped to 84.1% of the initial specific capacity in the fifth cycle, reaching only 505.8 mAh g^−1^. As previously discussed, this phenomenon is mainly attributed to the excessively high SSA of the EG, which significantly increases the contact between the active material and the electrolyte, thereby triggering intense secondary reactions during charge and discharge cycles [24]. These side reactions not only continuously consume the electrolyte but also induce uncontrolled growth of the SEI layer, leading to the irreversible consumption of active lithium and consequent capacity fade [41].

#### 3.2.3. Cycling Stability

**Figure 6:**
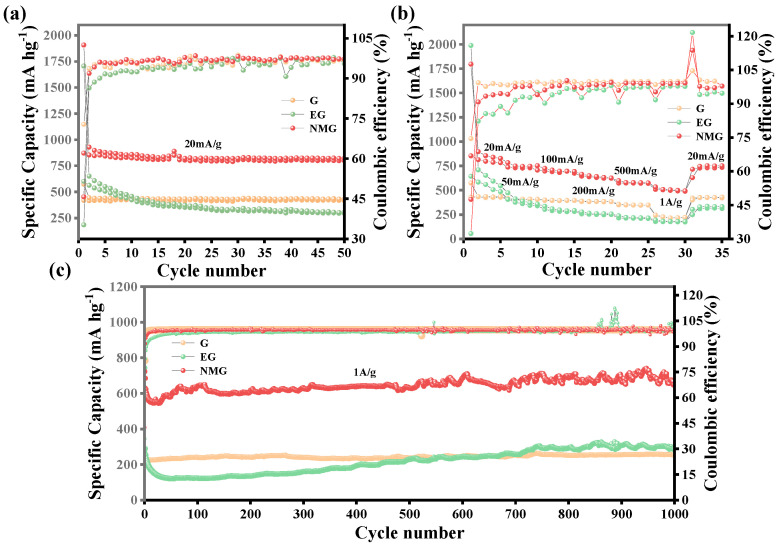
(**a**) Cycling performance at 20 mA g^−1^, (**b**) rate performance from 20 mA g^−1^ to 1 A g^−1^, (**c**) long cycles performance at 1 A g^−1^ of G, EG and NMG.

The cycling performance of the three electrodes over 50 cycles at a current density of 20 mA g^−1^ is compared in Figure 6a. After 50 charge–discharge cycles, the specific capacity of the EG declined sharply, retaining only 48.6% of the initial value, indicating poor cycle durability. In contrast, both of the NMG and the G demonstrated highly stable cycling behavior, with capacity retention rates of 91.5% and approximately 99.7%, respectively. Although the capacity retention of the NMG is slightly lower than that of the G, its reversible specific capacity after 50 cycles reaches 798.2 mAh g^−1^, nearly twice that of the G (417.2 mAh g^−1^). This performance indicates that the nitrogen-containing carbon framework in the NMG successfully reconciles high specific capacity with cycle stability, effectively overcoming the severe capacity fading observed in the EG during charging and discharging [42].

The rate performance of the three electrodes was evaluated at progressively increasing current densities ranging from 20 mA g^−1^ to 1 A g^−1^, with results shown in Figure 6b. At all current densities tested, the NMG exhibited the highest specific capacity. After five cycles at an initial low current density of 20 mA g^−1^, the NMG maintained a stable discharge capacity of 782.2 mAh g^−1^. As the current density gradually increased to 1 A g^−1^, its specific capacity showed a decreasing trend, which was attributed to increased overpotential and intensified diffusion limitation at high rates. Notably, when the current density was restored to 20 mA g^−1^ after 30 cycles, the NMG recovered a high reversible capacity of 734.8 mAh g^−1^, corresponding to a capacity recovery rate of approximately 93.9%. This excellent rate resilience fully demonstrates that the suitable interlayer spacing, porous structure and nitrogen-doped interface of the NMG effectively facilitate rapid ions/electrons even under high-rate cycling conditions.

The long-term cycling stability of the electrodes was further assessed at a high current density of 1 A g^−1^ over 1000 cycles (Figure 6c). The initial specific discharge and charge capacity of the NMG electrode are 1697.8 mAh g^−1^ and 723.1 mAh g^−1^ respectively. After 1000 cycles, the NMG still maintains a reversible capacity of 650.4 mAh g^−1^, substantially outperforming the G (266.2 mAh g^−1^). Remarkably, the capacity retention of the NMG reaches to 89.9% after 1000 cycles, demonstrating excellent cycling stability. In contrast, although the EG exhibits a higher initial capacity than of the G, its capacity decays rapidly and subsequently displays erratic fluctuations, ultimately stabilizing at 286.0 mAh g^−1^ after 1000 cycles.

This superior long-term stability of the NMG can be attributed to several synergistic factors. Firstly, the nitrogen-containing carbon layer formed on the NMG surface effectively prevents excessive volume expansion of the GO during microwave treatment, thereby ensuring structural integrity throughout prolonged cycling [43]. Secondly, the abundant oxygen-containing functional groups on the NMG surface, together with the carbon layer derived from the rapid thermal decomposition and carbonization of urea under microwave irradiation, provide additional sites for lithium-ion insertion, significantly enhancing the specific capacity [44]. Thirdly, the introduction of lithiophilic nitrogen atoms effectively reduces local charge transfer resistance and lowers the kinetic energy barrier of surface faradaic redox reactions [28]. This chemical modification accelerates interfacial dynamics, enabling rapid adsorption and diffusion at the electrode–electrolyte interface even under high-rate cycling conditions, consistent with the pseudocapacitive characteristics observed in the CV analysis. For comparative purposes, the electrochemical performance metrics of previously reported EG-based anode materials are summarized in Table 2, further contextualizing the superior performance of the NMG composite developed in this work.

#### 3.2.4. Electrochemical Impedance Spectroscopy

**Figure 7:**
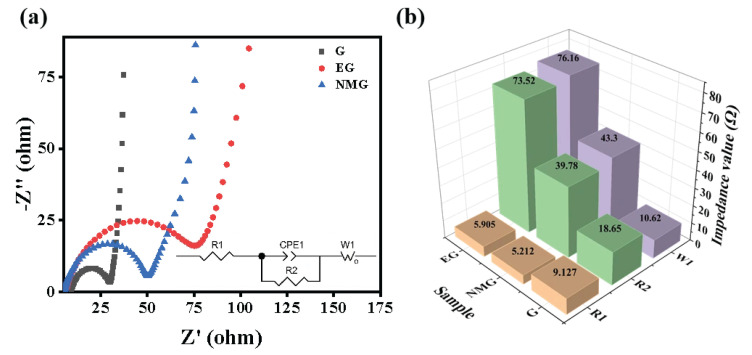
(**a**) EIS (R_1_ represents the internal resistance of the material itself, R_2_ represents the charge transfer internal resistance, and W_1_ represents the ion diffusion rate [52]) and (**b**) the corresponding comparison of impedance parameters for G, EG, and NMG.

To further elucidate the electrochemical kinetics and interfacial properties of the electrodes, EIS measurements were performed. Figure 7a presents the Nyquist plots of the G, EG, and NMG electrodes, while Figure 7b shows the corresponding comparison of impedance parameters. As shown in Figure 7a,b, the G electrode exhibits the smallest semicircle diameter in the high-frequency region and the steepest Warburg slope in the low-frequency region, indicating its intrinsically low charge transfer resistance and rapid ion diffusion capability. This favorable kinetics is attributed to the well-ordered graphitic structure with minimal surface defects, which facilitates efficient charge transfer and unhindered ion transport [50].

In contrast, the EG electrode displays the largest semicircle diameter and the most gradual Warburg slope, reflecting significantly higher charge transfer resistance and slower ion diffusion kinetics [51]. This impeded electrochemical behavior is primarily attributed to two interconnected factors. Firstly, the excessively high density of oxygen-containing functional groups on the EG surface hinders efficient charge transfer across the electrode–electrolyte interface [52]. Secondly, the excessively large specific surface area of the EG promotes more intense parasitic side reactions during electrochemical cycling, leading to the accumulation of resistive by-products at the interface. These accumulated species substantially impede ion migration, further deteriorating the electrochemical kinetics [19].

Notably, the NMG composite electrode demonstrates intermediate impedance characteristics, with a significantly reduced semicircle diameter compared to the EG and a Warburg slope approaching that of the G. This improved kinetic behavior indicates that, although the NMG shares some structural features with the EG, its suitable interlayer spacing, porous structure and nitrogen-doped active sites effectively mitigate the kinetic limitations encountered during charge–discharge cycling [53]. The nitrogen-doped carbon framework not only enhances electronic conductivity but also provides additional pathways for ion transport, while the optimized porous structure facilitates electrolyte penetration. Consequently, the NMG achieves a favorable balance, leveraging the benefits of expanded structure and heteroatom doping while avoiding the excessive interfacial resistance that plagues unmodified EG, thereby ensuring its excellent electrochemical performance.

#### 3.2.5. Electrochemical Kinetics

**Figure 8:**
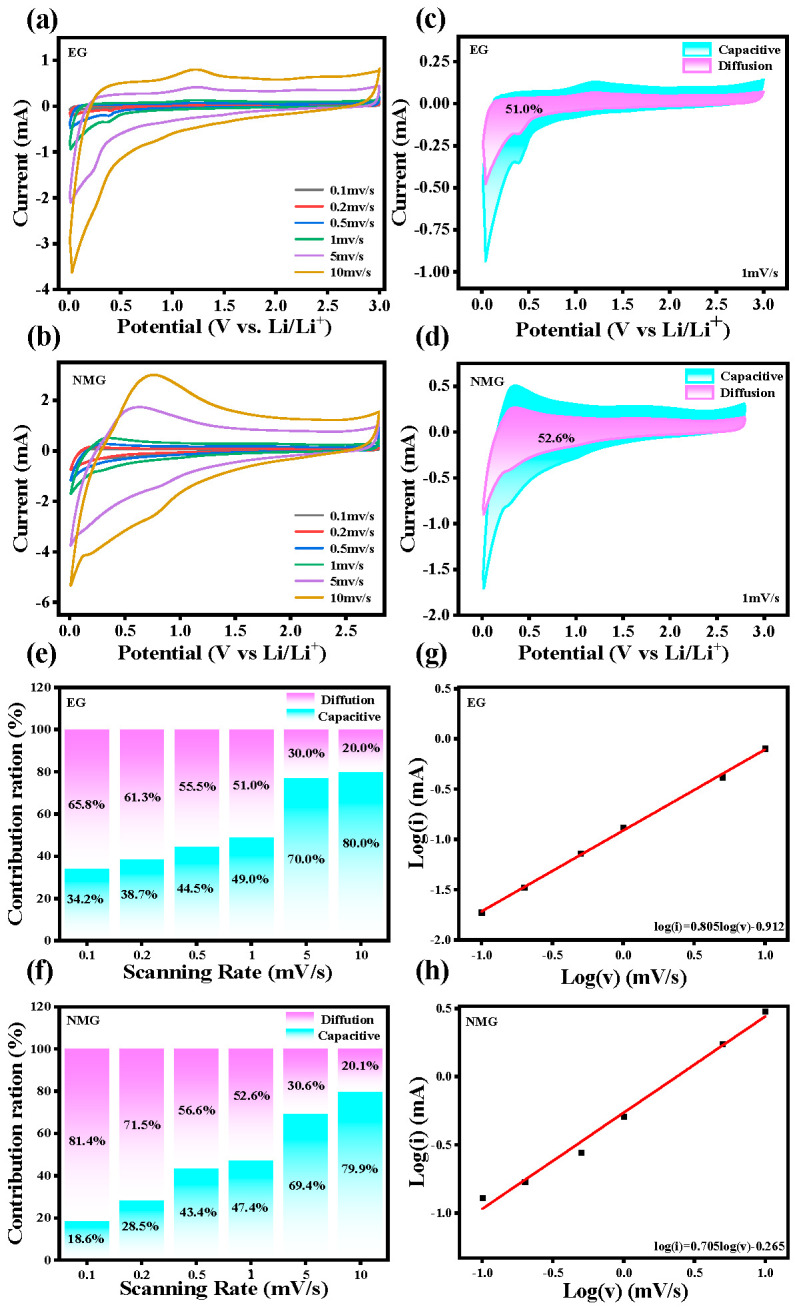
(**a**,**b**) CV curves of EG and NMG at various scan rates ranging from 0.1 to 10 mV s^−1^. (**c**,**d**) Pseudocapacitive contribution of EG and NMG at a scan rate of 1.0 mV s^−1^ (**e**,**f**) ratios of pseudocapacitive contributions for EG and NMG at different scan rates. (**g**,**h**) Linear relationship between *log i* and *log v* for EG and NMG.

To investigate the electrochemical kinetics of the electrode materials, the CV measurements were performed at various scan rates ranging from 0.1 to 10 mV s^−1^. As shown in Figure 8a,b, both of the EG and the NMG exhibit pseudo-capacitive energy storage behavior, with the CV profiles maintaining similar shapes as the scan rate increases, indicating good rate capability and electrochemical reversibility. The quantitative separation of capacitive and diffusion-controlled contributions was further analyzed using the method proposed by Wasinee et al. [54]. At a scan rate of 1.0 mV s^−1^, the pseudocapacitive contributions of EG and NMG are 49.7% and 52.6%, respectively. The slightly higher capacitive contribution of the NMG suggests that nitrogen doping enhances the surface-controlled charge storage process. As the scan rate increases from 0.1 to 10 mV s^−1^, the pseudocapacitive contribution exhibits a significant upward trend for both electrodes, while the diffusion-control fraction progressively diminishes. This evolution indicates that at higher scan rates, the charge storage mechanism gradually transitions towards a surface-dominated process, which is advantageous for fast charging applications [11]. At the highest scanning rate of 10 mV s^−1^, the pseudocapacitive contributions of EG and NMG reached 80.0% and 79.9% respectively, demonstrating excellent ionic conductivity and rapid charge–discharge capacity. Notably, the significant pseudocapacitive contribution observed in NMG aligns well with its excellent rate capability, confirming that surface-controlled capacitive behavior is one of the factors contributing to its high reversible capacity at high current densities [52,55]. The EG electrode exhibits a pseudocapacitive contribution similar to that of the NMG electrode, which contributes to its relatively high initial specific discharge capacity. Nevertheless, its excessively high SSA triggers severe parasitic side reactions during cycling, leading to rapid capacity fading and thus poor cycling stability [56].

## 4. Conclusions

In this study, nitrogen-containing micro-expanding graphite (NMG) composites were successfully constructed from waste graphite electrodes through a combined strategy involving chemical intercalation, microwave-assisted expansion, and in-situ nitrogen doping using urea as the nitrogen source. The nitrogen-doped amorphous carbon layer formed during microwave irradiation effectively moderates the expansion of graphite while preserving the characteristic worm-like hierarchical porous architecture, which provides multiple transmission channels for rapid ion transport and maintains structural integrity during electrochemical cycling. The XPS analysis confirms successful nitrogen incorporation with a predominant pyridinic-N configuration, introducing abundant defect sites and enhancing the lithiophilic nature of the carbon framework, with the nitrogen doping level and bonding configuration playing crucial roles in modulating the electronic structure and surface chemistry of the composite. Electrochemical evaluations demonstrate that the NMG electrode delivers exceptional lithium storage performance, including an initial discharge capacity of 1907.5 mAh g^−1^ at 20 mA g^−1^ and a reversible capacity of 798.2 mAh g^−1^ after 50 cycles—nearly double that of the G. Remarkably, even after 1000 cycles at a high current density of 1 A g^−1^, the NMG retains 650.4 mAh g^−1^ with a capacity retention of 89.9%, indicating an electrochemical activation process that progressively exposes additional active sites. Kinetic analysis reveals that the superior electrochemical performance originates from a synergistic interplay between diffusion-controlled intercalation and surface-dominated pseudocapacitance, with the nitrogen-doped defect sites and hierarchical pore architecture collectively promoting rapid ion/electron transport and facilitating surface faradaic reactions. This work establishes a viable pathway for the value-added upcycling of waste graphite electrodes, transforming an environmental burden into a high-performance anode material, while providing fundamental insights into the design of advanced carbonaceous anode materials through integrated defect engineering and heteroatom doping, offering a sustainable approach to meet the growing demand for next-generation LIBs.

## Figures and Tables

**Table 1:** Initial charge/discharge specific capacity and ICE of G, EG and NMG.

Sample	Discharge Specific Capacity (mAh g^−1^)	Charging Specific Capacity (mAh g^−1^)	ICE (%)
NMG	1907.5	872.5	45.7
EG	1705.8	601.0	35.2
G	574.5	422.2	73.5

**Table 2:** Electrochemical performance comparison of the EG-based anode.

Sample	Current Density	Reversible Capacity (mAh g^−1^)	Cycling Performance			Ref
			Current Density	Number of Cycles	Cycle Capacity (mAh g^−1^)	
NMG	0.02 A g^−1^	872.5	1 A g^−1^	1000	650.4	Our work
P@expanded-G50	0.1 A g^−1^	980	1 A g^−1^	500	510	[40]
EG	0.1 C	407.6	1 C	1000	347.7	[20]
BFAC@MEG	0.1 C	370	1 C	500	320	[45]
EG60	0.2 A g^−1^	277	1 A g^−1^	500	185	[46]
MEG-800	0.1 A g^−1^	462.1	0.1 A g^−1^	100	470.9	[47]
CEG-PANI (1:0.2)	0.2 A g^−1^	967.8	1 A g^−1^	200	400	[21]
EGC-Si	0.1 A g^−1^	709.1	1 A g^−1^	400	410	[48]
KG	0.2 A g^−1^	288.15	1 A g^−1^	300	160	[49]
EG	0.1 C	380.6	0.2 C	300	278	[19]

## Data Availability

The original contributions presented in this study are included in the article/Supplementary Material. Further inquiries can be directed to the corresponding author.

## Supplementary Materials

The following supporting information can be downloaded at https://www.mdpi.com/article/10.3390/nano16080485/s1, Preparation of Pure Graphite (G). Figure S1: Flow chart of preparation of G.
