# Supplementary material for: Fabrication of Nitrogen-Containing Micro-Expanding Graphite Composites from Waste Graphite Electrodes for Enhanced Lithium Storage
Source: Nanomaterials (Basel). 2026 Apr 19;16(8):485. doi: 10.3390/nano16080485 (PMC13118793; doi:10.3390/nano16080485)
Supplement: Supplementary file 1 [file nanomaterials-16-00485-s001.zip › nanomaterials-4240286-supplementary.pdf]

Preparation of Pure Graphite (G)

The G was prepared through a combined process of mechanical grinding and chemical purification. Initially, 10 g of waste graphite electrodes was loaded into a 250 mL stainless steel grinding jar containing 200 g of zirconia beads (5 mm in diameter). The milling process was performed in a planetary ball mill (YXQM-0.4L, Miqi MITR Instrument Co., Ltd., Changsha, Hunan Province) at 400 rpm for 48 h. Subsequently, 1 g of the as-milled powder was treated with 20 mL of a 40 wt% sodium hydroxide (NaOH) solution at 80 °C for 3 h to remove metallic and siliceous impurities. After thorough washing and drying, the material was further subjected to acid treatment using a 10 mL mixture of concentrated hydrochloric acid (HCl) and deionized water (v/v, 1:1) at 80 °C for 80 min. Finally, the product was washed with deionized water until the pH was neutral, and then it was vacuum-dried at 60°C for 12 hours to obtain G powder.

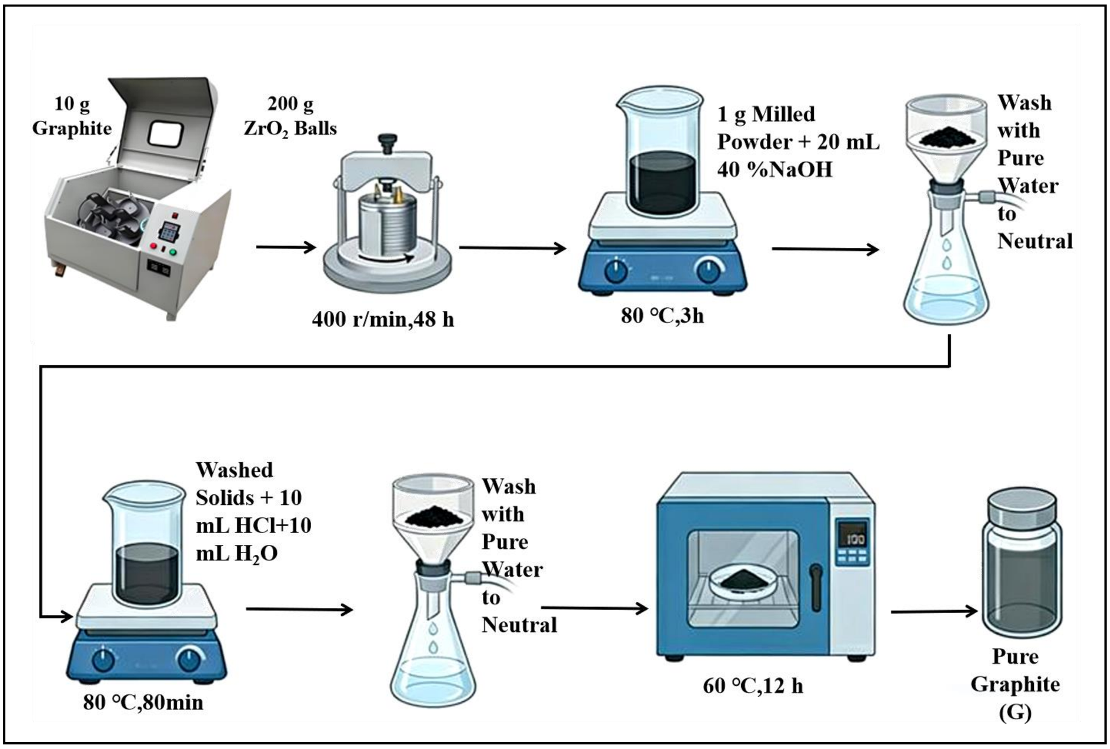

Figure S1 Flow chart of preparation of G
